# Interaction kinetics of peptide lipids-mediated gene delivery

**DOI:** 10.1186/s12951-020-00707-1

**Published:** 2020-10-17

**Authors:** Yinan Zhao, Tianyi Zhao, Yanyan Du, Yingnan Cao, Yang Xuan, Huiying Chen, Defu Zhi, Shutao Guo, Fangli Zhong, Shubiao Zhang

**Affiliations:** 1 Key Laboratory of Biotechnology and Bioresources Utilization of Ministry of Education, College of Life Sciences, Dalian Minzu University, Dalian, 116600 China; 2 School of Materials Science and Engineering, Zhengzhou University, Zhengzhou, 450001 China; 3 Key Laboratory of Functional Polymer Materials of Ministry of Education, State Key Laboratory of Medicinal Chemical Biology and Institute of Polymer Chemistry, College of Chemistry, Nankai University, Tianjin, 300071 China; 4 School of Chemistry and Pharmaceutical Engineering, Jilin Institute of Chemical Technology, Jilin, 132022 China

**Keywords:** Peptide lipids, Interaction kinetics, Uptake, Gene release, Gene delivery

## Abstract

**Background:**

During the course of gene transfection, the interaction kinetics between liposomes and DNA is speculated to play very important role for blood stability, cellular uptake, DNA release and finally transfection efficiency.

**Results:**

As cationic peptide liposomes exhibited great gene transfer activities both in vitro and in vivo, two peptide lipids, containing a tri-ornithine head (LOrn3) and a mono-ornithine head (LOrn1), were chosen to further clarify the process of liposome-mediated gene delivery in this study. The results show that the electrostatically-driven binding between DNA and liposomes reached nearly 100% at equilibrium, and high affinity of LOrn3 to DNA led to fast binding rate between them. The binding process between LOrn3 and DNA conformed to the kinetics equation: y = 1.663631 × exp (− 0.003427x) + 6.278163. Compared to liposome LOrn1, the liposome LOrn3/DNA lipoplex exhibited a faster and more uniform uptake in HeLa cells, as LOrn3 with a tri-ornithine peptide headgroup had a stronger interaction with the negatively charged cell membrane than LOrn1. The efficient endosomal escape of DNA from LOrn3 lipoplex was facilitated by the acidity in late endosomes, resulting in broken carbamate bonds, as well as the “proton sponge effect” of the lipid.

**Conclusions:**

The interaction kinetics is a key factor for DNA transfection efficiency. This work provided insights into peptide lipid-mediated DNA delivery that could guide the development of the next generation of delivery systems for gene therapeutics.
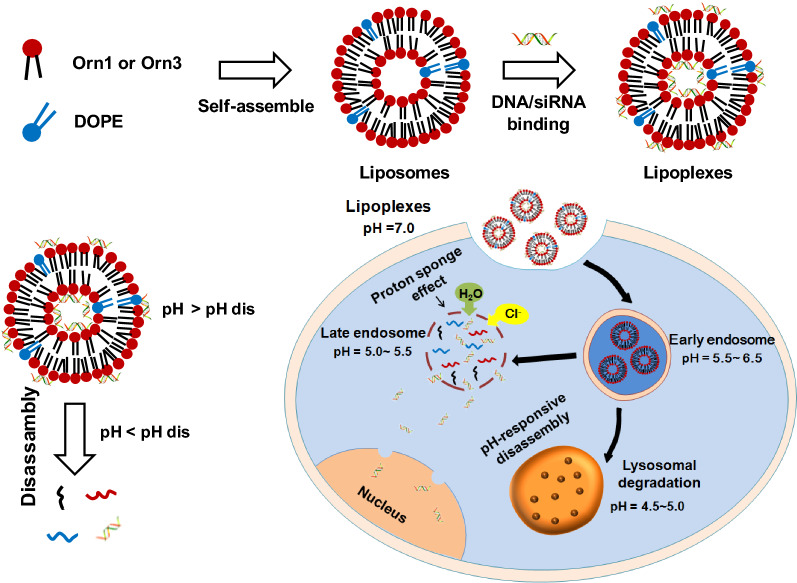

## Introduction

Gene therapy is the technology to transfer exogenous normal gene into the target cells to correct or compensate disease caused by genetic defects and anomalies, so as to achieve the aim of treating disease [1, 2]. Peptide-based cationic lipids are promising nonviral tools for nucleic acid delivery into cells, e.g., plasmids DNA for transfection and siRNA for gene silencing [3–6]. They have shown to possess properties that are superior to those of other cationic lipids, such as good biodegradability, excellent biocompatibility, and the ability to target to cells [7–10]. The primary requirements for a successful peptide lipid vector are the ability to compact nucleic acid, to protect it against degradation, and to deliver it into the cell with efficiency and specificity. In addition, liposome/DNA lipoplex must bind to the cell surface, enter cells by adsorption-mediated endocytosis, and accumulate in endosomes [11, 12]. Finally, lipoplexes escape from the endosomes and release the nucleic acid for successful gene expression [13–15].

In the process of delivering nucleic acid by peptide liposome vectors, the interaction between them has a very important effect on cellular uptake, nucleic acid release, and DNA or siRNA escape from endosomes, which are necessary for efficient gene transfer. Therefore, a perfect balance in the interaction between the liposomes and nucleic acid is required for the lipoplexes to behave as successful gene delivery vectors. Without knowledge of the interaction of liposomes with gene and the limiting barriers involved, it will be difficult to take a rational approach to develop improved methods of gene transfer and to test specific hypotheses related to cellular and molecular mechanisms.

**Fig. 1:**
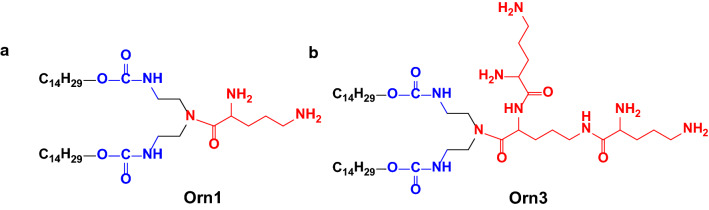
Structures of the peptide lipids (**a**) Orn1 and (**b**) Orn3

Researchers have used the model of quaternary ammonium lipid to corroborate the interaction between lipid and DNA. They found that the binding between lipids and DNA is accomplished by electrostatic attraction [16]. When DNA is mixed with liposomes composed of cationic lipids and helper-lipids, the resulting lipids/DNA lipoplexes may consist of a multilamellar strucutre (L^C^_α_) comprising DNA monolayers sandwiched between lipid bilayers. L^C^_α_ structure could be converted to an inverted hexagonal (H^C^_‖_) structure by adjusting the helper-lipid, then the H^C^_‖_ phase of lipids/DNA lipoplexes increase fusion of the lipoplex with the endosomal membranes in cells, and promote the DNA release into the cytoplasm, therefore increasing the transfection efficiency [17, 18]. However, systemic and thorough interaction research between peptide-based lipids and DNA has hardly been performed by far. To better understand the gene transport process and develop additional applications of peptide lipids, we here report on the use of 1,2-bis-myristyloxyamidopropyl tri-ornithine lipid (Orn3) for performing the research, and we used 1,2-bis-myristyloxyamidopropyl ornithine lipid (Orn1) as a control (their structures were shown in Fig. 1). Both were developed in our laboratory, and these lipids could efficiently transfer DNA and siRNA into tumor cells and tumors of mice, with little in vitro and in vivo toxicity [19].

**Fig. 2:**
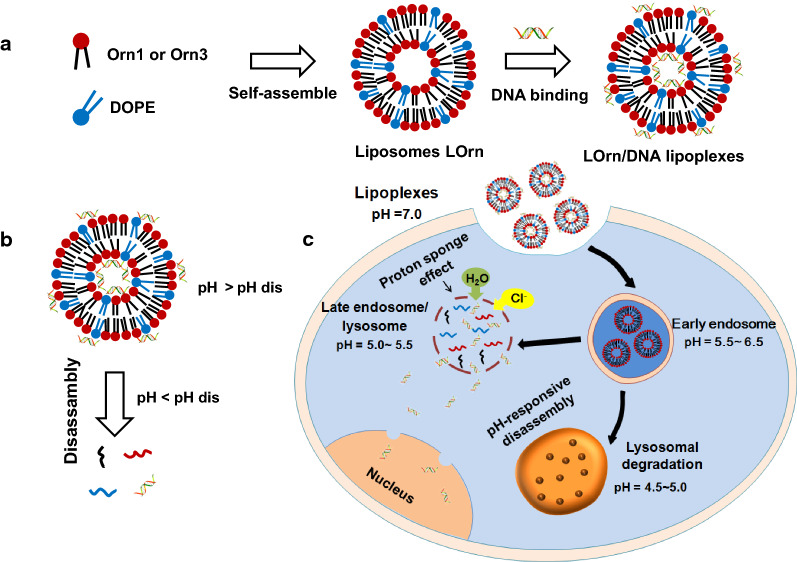
Assembling process of the liposome/DNA lipoplex and the intracellular transport of internalized DNA. **a** Self-assembly of a liposome and the interaction of a liposome with DNA. **b** Dissociation of a lipoplex. **c** Lipoplexes respond to the slightly acidic environment of early endosomes and release the cargoes

We examined the kinetics of association and dissociation between lipids and DNA. The cellular uptake of the lipoplexes at the microscopic level was further analyzed. Furthermore, endosomal escape of DNA as mediated by lipoplexes was detected by live cell imaging of HeLa cells to further clarify the mechanism of lipid-mediated delivery of DNA. As shown in Fig. 2, the peptide lipids self-assembled into liposomes, which could bind DNA by charge interactions (Fig. 2a). The lipid in liposome was composed of acid-responsive carbamate bond, lipoplexes could disassemble under disassembly pH (pH_dis_) (Fig. 2b). After uptake by the cells, the lipoplexes could stimulate endosome escape due to proton buffering capacity. DNA was then released from lipoplexes under the late endosomes environment (pH = 5.0 ~ 5.5), and efficient gene expression was obtained (Fig. 2c). In this process, we have found that the liposome LOrn3 with tri-peptide headgroup showed a significantly faster binding rate and higher affinity to DNA, and also showed higher efficiency for gene delivery. Hence, the revealed interaction kinetics of peptide lipids-mediated gene delivery in this study may serve guiding information in designing of gene carriers and studying mechanism of gene delivery.

## Materials and methods

### Materials

The peptide lipids Orn1 and Orn3 were synthesized by our lab. The co-lipid 1,2-Dioleoyl-3-trimetry-lammonium-propane chloride salt (DOPE) was purchased from Sigma-Aldrich (USA). The green fluorescent protein reporter gene (GFP-N1) was purchased from Promega Biotech Co. Ltd. (Beijing, China), and extracted in our lab. The labeled DNA with 6-carboxy-fluorescein (FAM) was purchased from GenPharma company (China). The DNA intercalating dyes GelRed (10,000 × in water), heparin, the liposome intercalating dye N-(7-Nitrobenz-2-Oxa-1,3-Diazol-4-yl)-1,2-Dihexadecanoyl-sn-Glycero-3-Phosphoethanolamine (NBD-PE), hoechst and Cy5 Nucleic Acid Labeling Kit were purchased from Biyotime biotechnology company (China), respectively. Trypsin was purchased from Gibco company (USA). All other chemicals were purchased from Alading (Shanghai, China). All water used was purified using a Milli-Q Plus 185 water purification system (Millipore, USA), giving a resistivity greater than 18 MΩ.

### Liposomes preparation and characterization

To prepare liposomes, peptide lipid and DOPE at the molar ratio of 1:1 were dissolved in 1 mL of chloroform in a glass vial; the solvent was removed under a stream of nitrogen gas, followed by high-vacuum desiccation. The dry lipid film was resuspended in 1 mL distilled water to give liposomes in a concentration of 1 mg/mL. Liposome solutions were subjected to several cycles of sonication in a bath sonicator and vigorous vortex mixing to form small vesicles. The average particle sizes and Zeta potential of liposomes were measured by dynamic light scattering (DLS, HORIBASZ-100, Japan). Twenty microliter of liposomes were diluted to 1 mL with distilled water, all measurements were performed with freshly prepared liposomes at 25 °C and in triplicate.

### Cell culture

The HeLa cell line was purchased from Stem Cell Bank, Chinese Academy of Sciences (Shanghai, China) and grown in Dulbecco's modified Eagle's medium (DMEM, Gibco, Inc., USA) media supplemented with 10% fetal bovine serum (Gibco, Inc.) and 1% antibiotic mixture. The cells were grown at 37 °C with 65% humidity.

### DNA transfection

To prepare lipoplexes, cationic liposomes were mixed with GFP-N1 in DMEM at liposome/DNA charge ratios ( ±) from 1:1 to 8:1 and incubated for 30 min at room temperature. In vitro DNA transfection of lipoplexes was measured against HeLa cell. The cells were seeded at 5 × 10^5^ cells/well in 24-well plates. After 24 h, medium was replaced with serum-free medium, and lipoplexes in medium were added to each well and incubated for 4 h at 37 °C. The medium was then replaced with medium containing 10% FBS and 1% antibiotics, and the cultures were maintained at 37 °C under 5% CO_2_. The expression of green fluorescent protein was measured by an inverted fluorescence microscope (Olympus IX71, Japan) and a flow cytometry (Becton–Dickinson, Heidelberg, Germany).

### Agarose gel electrophoresis

The interaction of peptide liposomes with DNA at different charge ratios was confirmed by gel retardation assay. The final volume of lipoplexes was adjusted to 20 µL, and then mixed with 6 × loading buffer (2 μL). The mixture was loaded onto a 1.2% agarose gel containing 3 μL GelRed. Electrophoresis was carried out in 1 × TAE running buffer at 90 V for 50 min, and DNA bands were visualised at UV light wavelength of 300 nm by a gel documentation unit (LongGene LG3000, China). The binding amounts of DNA by peptide liposomes (or DNA release rate) were calculated using software of gel documentation unit.

### Cytotoxicity of lipoplexes

Cytotoxicity of lipoplexes was analyzed by the MTT assay against HeLa tumor cells. Cells were seeded in 96-well plates and incubated at 37 °C under 5% CO_2_ for 18–24 h to achieve about 80–90% confluence. The LOrn1/DNA and LOrn3/DNA lipoplexes were added to the cells and incubated at 37 °C under 5% CO_2_ for 12, 24, 48 and 72 h, respectively. MTT (20 μL, 5 mg/mL, pH 7.4) was added, and cells were incubated with MTT for 4 h, following which 150 μL DMSO was added to each well to dissolve the substrate. The absorbance at 570 nm was monitored by Microplate Reader (Sunrise Tecan, Australia). Cells without lipoplexes served as controls. Cell viability was expressed as a percentage of the control. Cell viability was calculated as $\left( {\left[ {{\text{Abs}}} \right]_{{{\text{sample}}}} /\left[ {{\text{Abs}}} \right]_{{{\text{control}}}} } \right)\, \times \,{\text{1}}00\% .$

### Steady-state fluorescence spectroscopic of DNA binded with liposomes

To make the GelRed labeled DNA, the DNA stock solutions and GelRed stock solutions were mixed at the DNA and GelRed weight ratio of 10:1 in the phosphate buffer and equilibrated for 15 min. To make the liposomes/DNA lipoplexes, the peptide liposomes LOrn1 and DNA were mixed at 4:1 charge ratio ( ±), the ratio was 3:1 for LOrn3 and DNA. To a GelRed-DNA mixture in a quartz cuvette were added the desired amounts of peptide liposomes stock solutions. The excitation wavelength of GelRed was 300 nm, the emission was 600 nm. The excitation slit and emission slit were fixed at 1 and 0.8 nm, respectively. The temperature was set at 25 °C. Recorded the fluorescence intensity of system for every 5–10 min.

### Small-angle X-ray scattering (SAXS)

Small-angle X-ray scattering (SAXS) measurement was determined by an Anton Paar Saxesess mc2 instrument (Cu-Kα) (Anton Paar, Austria). The X-ray generator was operated at 40 kV and 50 mA. A sample-detector distance was 260 mm and the X-ray wavelength (λ) was 0.1542 nm. The sample was measured at 25 °C.

### Stopped-flow fluorescence spectroscopic of DNA binded with liposomes

The kinetic measurements were carried out using a stopped-flow fluorescence spectroscopic (SX-20MV, Applied Photophysics Ltd, UK). DNA (4 µg/mL) and GelRed (10,000 ×) were mixed and kept for 40 min to get DNA/GelRed complex. Liposomes (1 mg/mL) and DNA/GelRed complex at the charge ratios ( ±) of 3:1 or 4:1 were used for stopped-flow fluorescence spectroscopy, and the excitation monochromator was set to 510 nm. During the experiment, two separate syringes of the stopped-flow were filled up with DNA-GelRed complex and liposome solutions, and in each run, an equal volume of both solutions was injected at once into the sample chamber. The emission spectra were monitored continuously both before (t = 0 s) and after the injection.

### The release of DNA from lipoplexes with agarose gel electrophoresis

The lipoplexes were prepared with the method of DNA-binding assay above. The LOrn1/DNA and LOrn3/DNA lipoplexes were added to a given concentrations (0.05–2.0 µg/µL) of heparin dissolved in PBS and incubated for 30 min at room temperature [20]. The samples (20 µL) were electrophoresed on 1.2% agarose gel at different incubation time. Small amount of LOrn1/DNA and LOrn3/DNA lipoplexes were incubated with pH 5.5 phosphate buffer for 1, 4, 12, 24, 36, 48, 60 and 72 h, respectively, and the resulting solution was assessed by agarose gel electrophoresis.

### Cellular uptake using flow cytometry

Cellular uptake experiments were performed on HeLa cell line. LOrn1 and LOrn3 liposomes with the concentration range of 1.8 µg/mL were prepared (diluted by DMEM without FBS, pH 7.4). The liposome/FAM-DNA charge ratios ( ±) were 4:1 and 3:1 for LOrn1 and LOrn3, respectively. HeLa cells were seeded in 24-well plates at a density of 10^5^ cells/well and incubated in complete cell medium for 24 h. Then, the medium was removed and replaced with 400 µL DMEM per well without FBS. The lipoplexes (100 µL) were added and incubated at 37 °C for 4 h, the final concentration of LOrn1 and LOrn3 in lipoplexes were 8 μg/mL and 6 μg/mL, respectively. Cells were washed with PBS, trypsinized, and resuspended in PBS at a concentration of 1 × 10^6^ cells/mL. Then that used Trypan Blue to quench FAM-DNA fluorescence outside of cells, a stock solution of 0.4% (w/w). Trypan Blue in water was prepared and added at 10% of the volume of the cell suspension immediately before measurement. Cells were detected and quantified by flow cytometry (Becton–Dickinson, Heidelberg, Germany). Data were processed using the Cflow Plus software (Becton–Dickinson) [21, 22].

### Cellular uptake and DNA release using CLSM

Confocal laser scanning microscopy (CLSM) and TEM were used to investigate the intracellular transport of lipoplexes [23, 24]. Hoechst 33,342, NBD-PE and Cy5-DNA were used here in order to detect the intracellular distribution of liposome/DNA lipoplexes. The LOrn1 (or LOrn3) liposome was mixed with NBD-PE ethanol solution at liposome/NBD-PE volume ratio of 20:1, and stored overnight at 4 °C. Cy5-labeled DNA was prepared by use of the Label IT Cy5 Nucleic Acid Labeling Kit, according to the manufacturer's instructions. HeLa cells were seeded into 6-well plates, cells were transfected with liposome/DNA lipoplexes after 24 h. After 30 min, 2 h and 4 h of transfection, the treated cells were washed three times with 1 × PBS to remove residual free lipoplexes. Then 400 µL fresh DMEM containing Hoechst 33,342 was added into each well to stain cells. Hoechst 33,342 was used to stain cell nuclei. At last, the treated cells were imaged by CLSM (Olympus FV1000, Japan). For study of intracellular DNA release, LysoTracker Red was used to stain lysosomes [25]. Cells were exposed to LOrn3 liposome for 30 min, 2, and 4 h, cells were washed 3 times with PBS, fixed with 2.5% formaldehyde/2.5% glutaraldehyde in 0.1 M sodium cacodylate buffer at room temperature, and kept at 4 °C overnight. Then, the samples were washed several times in water, then dehydrated in increasing concentrations of ethanol, infiltrated, and embedded in Spurr's low-viscosity medium. The samples were polymerized in a 60 °C oven for 2 days. Ultrathin sections were cut in a Leica Ultracut microtome, stained with lead citrate in a Leica EM stainer (Leica, EM FC7 UC7, USA), and examined in a transmission electron microscope (JEM-2100, Carl Zeiss, Japan).

### Statistical analysis

All data were expressed as mean ± standard deviation (SD) for three independent tests. Differences between groups were analyzed using one-way analysis of variance (ANOVA) followed by LSD's multiple comparison post hoc test. The results were considered statistically significant when *p* < 0.05 (^*^p < 0.05; ^**^p < 0.01; ^***^p < 0.001).

## Results and discussion

### Characterization of liposomes

**Fig. 3:**
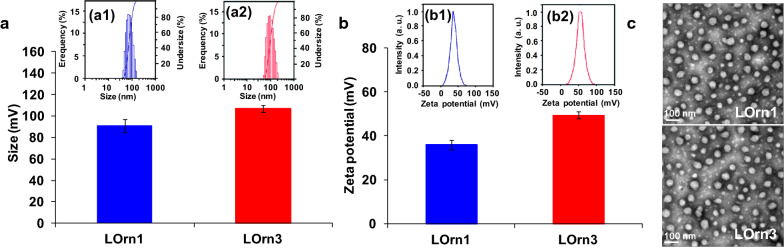
Characterization of liposomes. **a** Particle size and (**b**) Zeta potential of liposomes measured by using a ZetaSizer. Particle size distribution of LOrn1and LOrn3 is shown in (**a1**, **a2**), the PDI of LOrn1 and LOrn3 was 0.102 ± 0.014 and 0.097 ± 0.006, respectively. Zeta potential distribution of LOrn1 and LOrn3 is shown in (**b1**,** b2**). **c** Morphology of the particles measured by TEM, the diameters of Lorn1 and LOrn3 were 42.85 ± 16.3 nm and 44.16 ± 12.07 nm, respectively (voltage: 100 kV)

Our previous study showed that peptide liposomes with tri-ornithine as the headgroup (LOrn3) could transfer DNA and DNA very efficiently into tumor cells, such as NCI-H460 and Hep-2 cells. Also, it was able to deliver combined DNAs against c-Myc and VEGF for silencing distinct oncogenic pathways in lung tumors of mice, with little in vitro and in vivo toxicity [19]. To develop further applications of peptide lipids for gene delivery, a comparison with the lipid LOrn1, with one ornithine as the headgroup, was used to obtain detailed information about the interaction between peptide lipids and genes and the intracellular transport process. Our previous study showed that liposomes had the highest transfection efficiency with equivalent molar ratio of DOPE, accordingly the liposomes LOrn1 and LOrn3 were prepared with DOPE as a co-lipid at a molar ratio of 1:1. Figure 2a shows that the sizes were around 80 nm for both liposomes LOrn1 and LOrn3 (Fig. 3a). And the Zeta potentials of LOrn1 and LOrn3 were 30 mV and 45 mV respectively (Fig. 3b), which ensured the stability of the liposomes. The morphological characteristics were visualized by transmission electron microscopy (TEM) (Fig. 3c). Images obtained from LOrn1 and LOrn3 liposomes revealed well-defined spheres structures, regularly shaped and uniformly distributed.

### DNA transfection in vitro

**Fig. 4:**
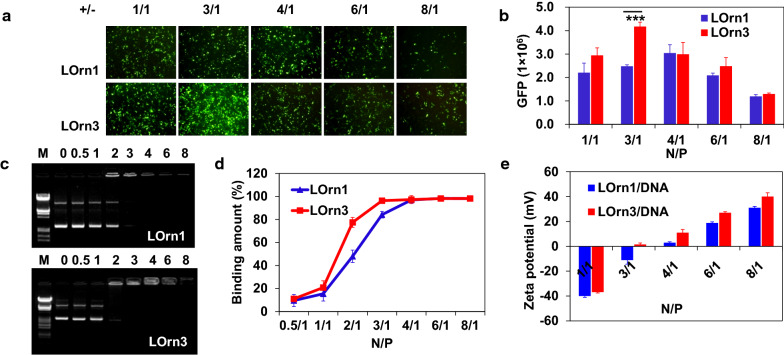
**a** Transfection efficiency of lipoplexes against HeLa cells. GFP expression of pGFP-N1 was mediated by liposomes LOrn1 and LOrn3 at the charge ratios ( ±) of 1:1, 3:1, 4:1, 6:1 and 8:1. The measurement was carried out in cells by using an inverted fluorescence microscope (10 × 10). **b** Transfection efficiency in Figure (**a**) was quantified by flow cytometry analysis. **c** Agarose gel electrophoresis of liposomes/DNA lipoplexes at various charge ratios. Line M: marker [λ DNA/EcoRI + HindIII]; Line 0: naked DNA [0.2 μg]; Lines 0.5–8: charge ratios were 0.5:1, 1:1, 2:1, 3:1, 4:1, 6:1 and 8:1. **d** Quantitative analysis by automatic analysis system of gel imaging. **e** Zeta potential of liposomes/DNA lipoplexes at charge ratios of 1:1, 3:1, 4:1, 6:1 and 8:1. Significance levels: ***p < 0.001

In our previous work, we reported that peptide liposomes delivered DNA and siRNA into various tumor cells with Lipofectamine 2000 as control. We discovered that the transfection efficiency of liposomes had a similar performance in HeLa, Hep-2, A549, MCF-7, H460 cells, though HeLa and Hep-2 cells showed higher transfection efficiency. Therefore, to evaluate further transfection efficiency of the two liposomes, we used them to deliver the GFP into HeLa and Hep-2 cells. The expression levels of GFP are shown in Additional file 1: Figure S1, Fig. 4a, b after the qualitative measurement of fluorescence and the quantitative determination of the fluorescent intensity. The charge ratios had a great impact on DNA transfection, LOrn1 and LOrn3 possessed the highest transfection efficiency at charge ratios of 4:1 and 3:1, respectively. And the expression of GFP of the LOrn3-treated cells was significantly stronger than that of the LOrn1-treated cells at the charge ratios from 1:1 to 8:1 in two cells. And the transfection efficiency against HeLa cell was higher than that against Hep-2 cells. Therefore, we chose HeLa cells to do the subsequent experiments. And it's noted that LOrn1/DNA and LOrn3/DNA lipoplexes had much lower toxicity to HeLa cells at charge ratios of 4:1 and 3:1, respectively. The cell viability was also over 80% after treatment with lipoplexes for 72 h (Additional file 1: Fig. S2).

Then, we investigated the ability of liposomes to complex DNA at charge ratios from 0.5:1 to 8:1, findings in the gel retardation assay revealed that DNA binding amount increased with the charge ratios increase. LOrn1 and LOrn3 completely bound DNA at charge ratios more than 4:1 and 3:1, respectively (Fig. 4c, d). Meanwhile, the Zeta potentials of lipoplexes were detected, the results show that their Zeta potentials increased from negative to positive with the increase of charge ratios (Fig. 4e). For LOrn1 and LOrn3, the values became positive just at charge ratios of 4:1 and 3:1, respectively, corresponding with the charge ratios of complete DNA complex, these results accorded well with the above gel retardation assay results. This study indicates that LOrn3 was more effective than LOrn1 in terms of the interaction with DNA. In addition, synchrotron small-angle x-ray scattering (SAXS) was used to analyze the structures of the liposome/DNA lipoplexes, and the liposome was composed of peptide lipid (LOrn1 or LOrn3) and DOPE at ratio of 1:1. For LOrn1/DNA and LOrn3/DNA lipoplexes, though the L^C^_α_ and H^C^_‖_ structures coexisted (Additional file 1: Fig. S3), LOrn3/DNA lipoplex formed more H^C^_‖_ structures than LOrn1/DNA, which was considered as one reason for higher transfection efficiency of LOrn3. SAXS scans showed sharp peaks at *q*_100_ = 0.106 Å^−1^ and *q*_200_ = 0.239 Å^−1^ from the lamellar periodic structure (*d* = 2*π*/*q*_100_ = 59.27 Å) and at *q*_10_ = 0.126 Å^−1^ from hexagonal structure. The peptide lipid-DOPE bilayer thickness was *d*m = 40 Å [17], the water gap between bilayer was 19.27 Å (*d*w = *d*–*d*m). While the hexagonal lattice constant *a* is 57.58 Å (*a* = 4*π*/(3)^0.5^*q*_10_). The middle broad peak at *q*_11_ = 0.186 Å^−1^ was due to the one-dimensional array of DNA chains, with the spacing between the DNA strands (*d*_DNA_ = 2*π*/*q*_11_), which demonstrated that the DNA was able to be inserted between cationic lipid bilayers (thickness is *d*w). During this process, DOPE is a fusogenic phospholipid that presents a super synergistic effect when used with cationic liposomes, decreasing the cytotoxicity imposed by the cationic lipids, destabilizing lipid bilayers by increasing the fusion with the cellular and endosomal membranes and allowing the release of DNA into the cytosol. Lipoplexes containing DOPE may facilitate the transition from lamellar to hexagonal structures promoting high gene transfection efficiency [17].

### Binding kinetics of liposome with DNA

**Fig. 5:**
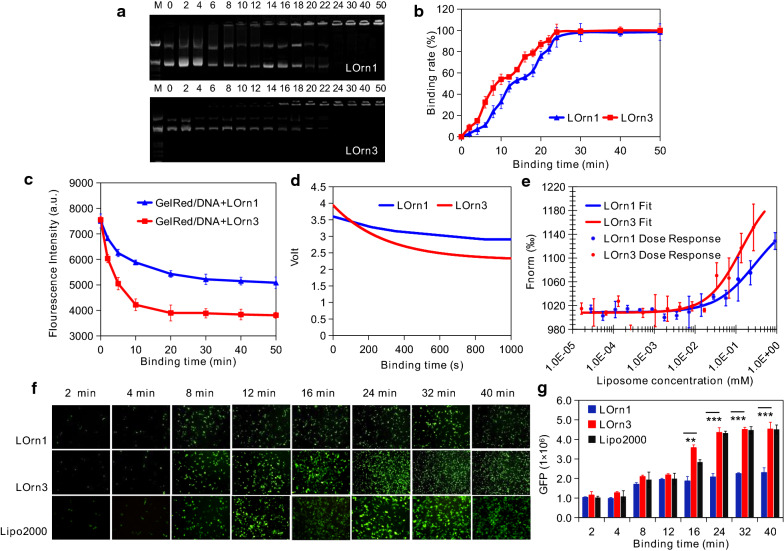
Kinetic traces for binding of peptide liposome and DNA. **a** Agarose gel electrophoresis of the lipoplexes at charge ratios of 4:1 and 3:1 for lipids LOrn1 and LOrn3, respectively (Line M: marker [λ DNA/EcoRI + HindIII]; Line 0: naked DNA [0.2 μg]; Lines 2–50: binding times were 2, 4, 6, 8, 10, 12, 14, 16, 18, 20, 22, 24, 30, 40 and 50 min). **b** Quantitative analysis by automatic analysis system of gel imaging. **c** Fluorescence intensity of labeled DNA with GelRed and liposomes bound to DNA at charge ratio ( ±) 3:1, at several interaction times: 0, 2, 5, 10, 20, 30, 40, and 50 min. **d** Stopped-flow fluorescence intensity decay of LOrn1/DNA and LOrn3/DNA lipoplexes binding at the charge ratios of 4:1 and 3:1, respectively. The final DNA and GelRed concentrations were 2 µg/mL and 0.2 µL/mL, respectively. The fluorescence was measured by excitation at a wavelength of 260 nm. **e** Affinity of liposomes to DNA, K_d_ values of LOrn1 and LOrn3 were 0.296 and 0.136 µM, respectively. All values are expressed as mean ± SD (n = 5). **f** Effect of liposome-DNA binding time on the transfection efficiency of liposomes with 2, 4, 8, 12, 16, 24, 32 and 40 min. **g** Quantitative analysis in Figure (f) by flow cytometry. All values are expressed as mean ± SD (n = 5). The amount of pGFP-N1 was 1 µg/well for 24-well plates. Lipo2000 was used as control. Significance levels: **p < 0.01, ***p < 0.001

To obtain detailed information about the interaction between liposomes and DNA, we utilized agarose gel electrophoresis and fluorescent resonance spectroscopy to examine the time-dependent interaction of cationic peptide liposomes with DNA. As shown in Fig. 5a, b, the binding amount of DNA significantly increased with time, and nearly 100% DNA binding was reached after 30 min and 24 min for LOrn1 or LOrn3 liposomes, respectively. The data also demonstrated that the binding speed between liposome LOrn3 and DNA was faster than that between LOrn1 and DNA. The interaction between liposomes and DNA labeled with a nucleic dye (GelRed) was also monitored by fluorescence spectroscopy [26, 27]. GelRed fluoresces strongly when bound to DNA, and the displacement of GelRed from DNA by liposomes results in a decrease in fluorescence intensity that correlates with the amount of liposome binding DNA. The results show that the displacement of GelRed from DNA by liposome LOrn3 was much faster than that by LOrn1 (Fig. 5c). Moreover, the fluorescence intensity of GelRed/DNA was lower for LOrn3 when the displacement reached the equilibrium. The results substantiate the fact that the DNA binding of liposomes is faster at the initial stage, and LOrn3 has a stronger DNA binding ability than LOrn1. To further investigate the binding process of the two liposomes and DNA, a stopped-flow fluorimeter (SX-20MV, UK) setup was employed to monitor the formation of liposome/DNA lipoplexes using the fluorescent probe GelRed. The data indicate that GelRed fluorescence decreased much more rapidly for LOrn3 than LOrn1 (Fig. 5d), and the K values of LOrn1 and LOrn3 were 0.102795 and 0.003472, respectively. By fitting the binding data, it was found that the binding process between liposomes and DNA conformed to the kinetics equations: y = 0.235141 × exp (-0.102795x) + 3.461248 and y = 1.663631 × exp (− 0.003427x) + 6.278163 for LOrn1 and LOrn3, respectively. It means that the binding rate of LOrn3 and DNA was faster than that of LOrn1 and DNA. Then the binding affinity of liposomes to DNA was identified by using MicroScale Thermophoresis (MST) performed on the Monolith NT.115Pico system. In this method, the affinity of liposome to DNA is represented by K_d_ value, which is the liposome concentration required to bind 50% DNA [28, 29]. The dose response curves generated by titrating LOrn1 or LOrn3 liposomes to DNA solutions (Fig. 5e) show that the K_d_ values of LOrn1 or LOrn3 were 0.296 and 0.136 µM, respectively. Therefore, we speculate that high affinity of LOrn3 to DNA led to fast binding rate between them, and then resulting in more efficient transfection. Therefore, we further evaluated the effect of interaction kinetics between the liposomes and DNA on the transfection efficiency. The expression of GFP gradually increased with binding time, and then reached to the maximum values at about 24 min and 32 min for LOrn3 and LOrn1 liposomes, respectively (Fig. 5f, g). At this time the interaction between liposomes and DNA also reached equilibrium (Fig. 5b, c). We confirmed that the interaction kinetics is a key factor for DNA transfection efficiency.

### Stability of lipoplexes in blood mimicked environment

**Fig. 6:**
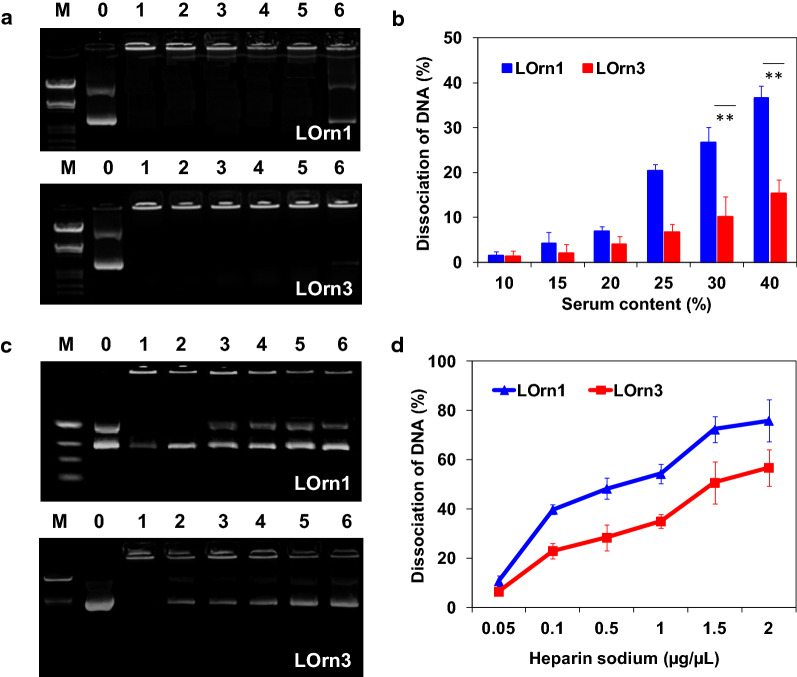
Effect of serum and heparin sodium on the stability of LOrn1/DNA lipoplexes with a charge ratio of 4:1 and LOrn3/DNA lipoplexes with an charge ratio of 3:1. **a** Agarose gel electrophoresis of the lipoplexes at different serum contents (Lane M: marker; Lane DNA: free DNA; Lanes 1–6: serum contents of 10%, 15%, 20%, 25%, 30%, and 40%, respectively). **b** Quantitative analysis of the dissociation rate of lipoplexes in the present of serum. **c** Agarose gel electrophoresis of the lipoplexes at different concentrations of heparin sodium (Lane M: marker; Lane DNA: free DNA; Lanes 1–6: heparin sodium concentrations of 0.05, 0.1, 0.5, 1.0, 1.5, and 2.0 µg/µL, respectively). **d** Quantitative analysis of the dissociation rate of lipoplexes in the present of heparin sodium. Each bar represents the mean ± SD (*n* = 5). Significance levels: **p < 0.01

Blood is rich in negatively charged macromolecules, such as serum protein and heparin, and these materials are known to dissociate DNA from lipoplexes by the interaction between liposomes and serum proteins [30–33]. Therefore, we first examined the effect of serum on the dissociation of lipoplexes, as shown in Fig. 6a, b, which depicts DNA release of lipoplexes in a medium with varying serum concentrations. A serum concentration of 10% had nearly no obvious effect on the stability of both lipoplexes, with a DNA release of about 2%. With increasing serum concentrations, the stability of both lipoplexes decreased, and the LOrn1/DNA lipoplex showed much more DNA dissociation than the LOrn3/DNA lipoplex. When the serum concentrations were greater than 25%, the release of DNA from the LOrn1/DNA lipoplexes increased to 20–35%; in contrast, the percentage of DNA released from the LOrn3/DNA lipoplexes under the same conditions was much lower. Our previous study had already revealed that lower concentrations of serum (5% or 10%) resulted in only a small reduction in transfection efficacy; with 20% serum, the transfection efficacy was slightly lowered [34]. Therefore, we hypothesize LOrn3 could well protect DNA in the lipoplexes from the interaction with serum when they are in the bloodstream.

Heparin sodium can competitively bind to liposomes from lipoplexes, which may lead to the dissociation of lipoplexes in blood. Therefore, the stability of lipoplexes was further examined by adding heparin sodium to simulate the blood environment. Figure 6c, d showed that the brightness and the area of the bands corresponding to free plasmid DNA increased with the increase in heparin concentration. Heparin sodium affected the stability of the LOrn1/DNA lipoplex to a much greater extent than the LOrn3/DNA lipoplex in the concentration range of 0.1–2.0 µg/µL. At a heparin concentration that is common in the blood (0.1 µg/µL), the release of DNA from the LOrn1/DNA lipoplex was 40%, and it was 20% for the LOrn3/DNA lipoplex. Therefore, the LOrn3/DNA lipoplex could show great advantages over LOrn1/DNA when used for gene delivery.

### Cellular uptake of lipoplexes

**Fig. 7:**
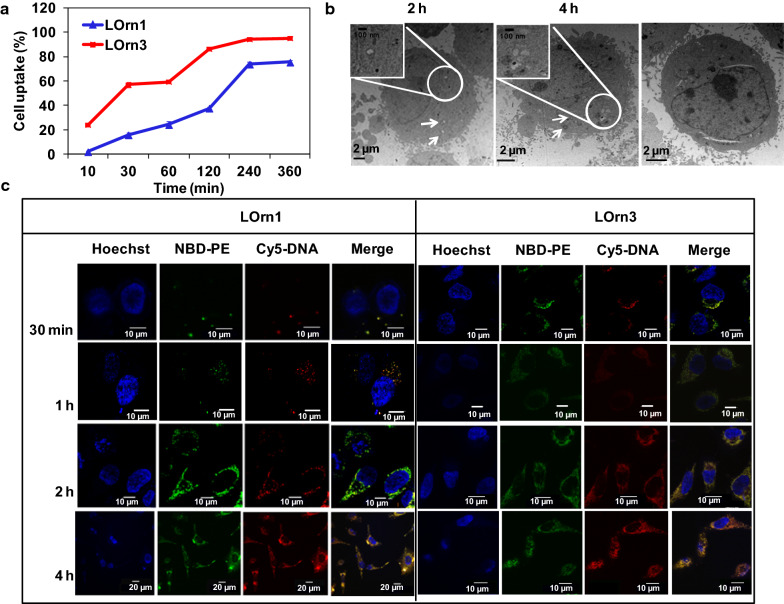
**a** Cellular uptake of liposome/FAM-DNA lipoplex in HeLa cells was evaluated by FACS. **b** Intracellular trafficking of LOrn3/DNA lipoplex in HeLa cells at incubation time of 30 min, 2 h, and 4 h by TEM. **c** Intracellular tracking of lipoplexes with incubation time of 30 min, 1 h, 2 h, and 4 h in HeLa cells by LSCM. Hoechst was used to label cell nucleus (blue), NBD-PE to label liposomes (green), and Cy5 to label DNA (red)

Cellular uptake is required for successful transfection of genes. The cellular uptake of liposomes/FAM-DNA lipoplexes was first evaluated by FACS within 6 h. As Trypan Blue could quench FAM-DNA fluorescence outside cells, it was used to differentiate internalized vs extracellular fluorescence markers. As displayed in Fig. 7a, the cellular uptake increased gradually with the transfection time; cellular uptake rates of LOrn1 and LOrn3 were about 73% and 94% after 4 h, respectively. LOrn3 showed a stronger interaction with the negatively charged cell membrane than LOrn1, so the LOrn3/DNA lipoplex could more easily cross the cell membrane, resulting in a higher cellular uptake rate compared with LOrn1/DNA. And we examined its morphological features at the ultra-structural level in HeLa cells incubated with LOrn3/DNA lipoplexes for different periods of time (Fig. 7b). The majority of LOrn3/DNA lipoplexes were found within early endocytic structures (endosomes) and multi-vesicular bodies in the early phase of uptake (2 h). They were detected within lysosomes 4 h after exposure of cells to LOrn3/DNA lipoplexes.

To further explore the kinetics of LOrn3/DNA lipoplex uptake, cellular uptake of the liposome/DNA lipoplexes was also investigated by laser scanning confocal microscope (LSCM) (Fig. 7c). As illustrated in the merged images, LOrn3/DNA lipoplexes displayed visible signals in the cytoplasm at 30 min post-transfection, but only a small amount of LOrn1/DNA lipoplexes were internalized into the cells. Though more LOrn1/DNA lipoplexes were localized in the cytoplasm at 2 and 4 h post-transfection, it was found that the fluorescent signals of LOrn3/DNA lipoplexes were distributed throughout the entire cytoplasm at 2 h post-transfection and mainly gathered around the nuclei at 4 h post-transfection. Compared to liposome LOrn1, liposome LOrn3/DNA lipoplexes exhibited a faster and more uniform uptake in HeLa cells. And combined with the interaction data of liposome and DNA (Fig. 5), it's found that the more the liposome-DNA interaction was, the higher the cellular uptake of liposome/DNA lipoplex was.

### Dissociation kinetics of liposome and DNA

**Fig. 8:**
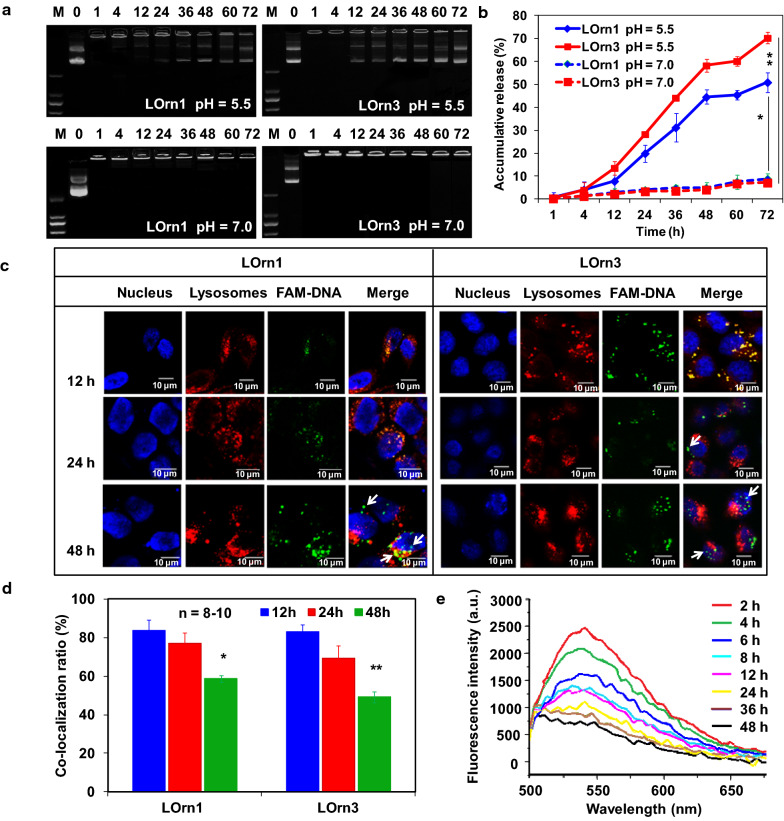
**a** DNA dissociation profile of the LOrn1/DNA and LOrn3/DNA lipoplexes, evaluated by gel electrophoresis at a pH of 5.5; the lipoplexes were measured at a pH of 7.0 as controls. **b** Quantitative analysis of accumulative DNA release by gel electrophoresis. Significance levels: *p < 0.05, **p < 0.01. **c** Intracellular release of DNA in HeLa cells as determined by LSCM. White arrows showed the aggregation state of FAM-labeled DNA. **d** Co-localization ratio of FAM-DNA (green) and lysosomes (red). 8–10 Cells in each group were selected randomly. Significance levels: *p < 0.05 vs. 12 h, **p < 0.01 vs. 12 h. **e** Fluorescence spectra of Rhodamine 123 in HeLa cell lysates with culture times of 2, 4, 6, 8, 12, 24, 36, and 48 h after transfection with LOrn3/DNA lipoplex

After the assembly with DNA, the stimuli-responsive features and DNA dissociation behavior of the lipoplexes were tested in vitro in a simulated intracellular microenvironment (pH 5–5.5) using a gel retardation assay [35, 36]. As can be seen in Fig. 8a, b, the DNA dissociated from lipoplexes was time-dependent at pH 5.5, and the DNA dissociation rate of the LOrn3/DNA lipoplex was faster than that of the LOrn1/DNA lipoplex. Seventy percent of DNA could dissociate from LOrn3/DNA lipoplex and only 51% from LOrn1/DNA lipoplex at 72 h. In contrast, little DNA dissociated from the two lipoplexes at the similar time point (8% at 72 h) at pH 7.0. The pH-induced structural destruction of lipoplexes contributed to the DNA release by exposing carbamate bonds in the lipids to the reductive environment for cleavage (Additional file 1: Fig. S4).

A major intracellular barrier for DNA delivery is endosomal entrapment followed by trafficking and lysosomal degradation or exocytosis [37, 38]. The escape of DNA from late endosomes/lysosomes into the cytosol is thought to be a rate-limiting step for many delivery approaches [39]. The lipoplexes are designed to facilitate late endosomal/lysosomal escape of DNA by synergistic effects of the proton sponge effect [40, 41] and the degradation of the peptide lipid LOrn1 or LOrn3 by carbamate bond breaks in late endosomes/lysosomes. To assess intracellular DNA release, confocal microscopy was used to investigate the distribution of FAM-labeled DNA (green) in lipoplexes and LysoTracker Red labeled late endosomes and lysosomes against HeLa cells. As shown in Fig. 8c, we observed that DNA was gradually released from the both of lipoplexes over time. For LOrn3/DNA lipoplex, most FAM signals of the LOrn3/DNA lipoplex were merged in late endosomes/lysosomes at 12 h, as shown by the yellow color. Much DNA could be released from late endosomes/lysosomes at 24 h after the transfection, and at 48 h, strong green fluorescence in the images for LOrn3/DNA lipoplex showed an obvious release of DNA from late endosomes/lysosomes (indicated with white arrows).

The co-localization ratio (%) is a commonly used index to measure DNA release [42, 43], which was carried out by the following formula:

$$ {\text{Co}} - {\text{localization}}{\mkern 1mu} {\text{ratio}}\left( \%  \right){\mkern 1mu} {\text{ = }}{\mkern 1mu} \frac{{{\text{Signal}}_{{{\text{yellow}}}} }}{{{\text{Signal}}_{{{\text{yellow}}}} {\text{ + }}{\mkern 1mu} {\text{Signal}}_{{{\text{green}}}} }} \times {\text{100}} $$

Signal_yellow_ means the fluorescence signal of FAM-DNA (green) co-localized with lysosomes (red), and Signal_green_ means the fluorescence signal of FAM-DNA in cytoplasm. A low co-localization ratio means quick DNA release from the late endosomes/lysosomes and a better distribution of the DNA in cytoplasm. The results show that the co-localization ratio gradually decreased with time; they were 49% and 58% at 48 h for LOrn3 and LOrn1, respectively (Fig. 8d). According to the results of the lipoplexes dissociation study in a simulated tumor microenvironment (Fig. 8b), the response to the acidic environment was probably caused by the break of carbamate bonds in the lipids. In addtition, DNA release of LOrn3/DNA lipoplex was more than that of LOrn1/DNA lipoplex at 48 h, this was consistent with the result in Fig. 8b. Although LOrn1 and LOrn3 have a similar chemical constitution, LOrn3, containing more amino groups, promoted not only cellular internalization [44] but also displayed a higher proton buffering capacity. Therefore, LOrn3/DNA lipoplex showed an enhanced capability in late endosomes/lysosomes escape and DNA release compared to LOrn1/DNA lipoplex. That also explains the reason why LOrn3 had more efficient transfection than LOrn1. Accordingly, FRET was utilized to further confirm the intracellular disassembly process of DNA from the LOrn3/DNA lipoplex in HeLa cells, by using Rhodamine-DNA and NBD-PE-LOrn3 liposome to constitute a Rhodamine/NBD FRET system (acceptor/donor) [45]. After the HeLa cells were transfected with lipoplexes for 4 h, they were incubated for 2, 4, 6, 8, 12, 24, 36, and 48 h. Figure 8e shows the fluorescence emission intensity of Rhodamine-labeled DNA decreased with time. The data indicate that less resonance energy was transferred from donor to acceptor due to lipoplexes dissociation in HeLa cells, and much of the DNA was released from lipoplexes at 48 h after transfection.

Together, these results strongly demonstrated that both of the lipoplexes could exist in a comparatively stable structure under normal physiological conditions (pH 7.0), while they rapidly release a significant amount of DNA due to the destabilization resulting from the acidic microenvironment in endosomes or lysosomes of tumor cells (pH 5.5) [46, 47].

## Conclusions

Our study provides insights into the interaction kinetics, cellular uptake, and dissociation of peptide liposome-mediated gene delivery. It highlights similarities and differences between liposomes with either tri-ornithine or mono-ornithine headgroups. We demonstrate that the peptide lipid with three ornithine headgroup (LOrn3) had a strong binding ability to DNA. Moreover, LOrn3/DNA lipoplex exhibited excellent intracellular stability, effective cellular uptake, lysosomal evasion, and intracellular gene release. Most importantly, we have illustrated the relationship between interaction kinetics and transfection efficiency. In summary, the study proposes a theoretical framework for strategies to improve gene delivery systems.

## Supplementary information

**Additional file 1: Figure S1.** (a) Transfection efficiency of lipoplexes against Hep-2 cells. GFP expression of GFP-N1 was mediated by liposomes LOrn1 and LOrn3 at the N/P ratios of 1:1, 3:1, 4:1, 6:1 and 8:1. The measurement was carried out in cells by using an inverted fluorescence microscope (10×10). (b) Transfection efficiency in Figure (a) was quantified by flow cytometry analysis.** Figure S2**. Cell viability assay of HeLa after treating with LOrn1/DNA and LOrn3/DNA lipoplexes for different time by MTT.** Figure S3**. Small angle x-ray scattering patterns of the lamellar LCα and columnar inverted hexagonal HC‖ phases of (a) LOrn1/DNA and (b) LOrn3/DNA lipoplexes at N/P ratios of 4:1 and 3:1, respectively.** Figure S4**. Effect of acid environment on lipid structure was detected by ESI-MS. (a) ESI-MS analysis of lipid under the pH of 7.0, (b) ESI-MS analysis of lipid under the pH of 5.5.

## Data Availability

All data generated or analyzed during this research are included in this article.
