# Supplementary material for: Interaction kinetics of peptide lipids-mediated gene delivery
Source: J Nanobiotechnology. 2020 Oct 17;18:144. doi: 10.1186/s12951-020-00707-1 (PMC7568367; doi:10.1186/s12951-020-00707-1)
Supplement: Supplementary file 1 — Additional file 1: Figure S1. (a) Transfection efficiency of lipoplexes against Hep-2 cells. GFP expression of GFP-N1 was mediated by liposomes LOrn1 and LOrn3 at the N/P ratios of 1:1, 3:1, 4:1, 6:1 and 8:1. The measurement was carried out in cells by using an inverted fluorescence microscope (10×10). (b) Transfection efficiency in Figure (a) was quantified by flow cytometry analysis. Figure S2. Cell viability assay of HeLa after treating with LOrn1/DNA and LOrn3/DNA lipoplexes for different time by MTT. Figure S3. Small angle x-ray scattering patterns of the lamellar LCα and columnar inverted hexagonal HC‖ phases of (a) LOrn1/DNA and (b) LOrn3/DNA lipoplexes at N/P ratios of 4:1 and 3:1, respectively. Figure S4. Effect of acid environment on lipid structure was detected by ESI-MS. (a) ESI-MS analysis of lipid under the pH of 7.0, (b) ESI-MS analysis of lipid under the pH of 5.5. [file 12951_2020_707_MOESM1_ESM.docx]

Additional file 1

Interaction Kinetics of Peptide Lipids-Mediated Gene Delivery

Yinan Zhao^1^, Tianyi Zhao^2^, Yanyan Du^1^, Yingnan Cao^1^, Yang Xuan^1^, Huiying Chen^1^, Defu Zhi^1^, Shutao Guo^3*^, Fangli Zhong^4*^, Shubiao Zhang^1*^

^1^ Key Laboratory of Biotechnology and Bioresources Utilization of Ministry of Education, College of Life Sciences, Dalian Minzu University, Dalian 116600, China

^2^ School of Materials Science and Engineering, Zhengzhou University, Zhengzhou 450001, China

^3^ Key Laboratory of Functional Polymer Materials of Ministry of Education, State Key Laboratory of Medicinal Chemical Biology and Institute of Polymer Chemistry, College of Chemistry, Nankai University, Tianjin 300071, China

^4^ School of Chemistry and Pharmaceutical Engineering, Jilin Institute of Chemical Technology, Jilin 132022, China

* Correspondence: zsb@dlnu.edu.cn; [stguo@nankai.edu.cn;](mailto:stguo@nankai.edu.cn;) [fanglizhong@sina.com](mailto:fanglizhong@sina.com)

**Figure S1.** (a) Transfection efficiency of lipoplexes against Hep-2 cells. GFP expression of GFP-N1 was mediated by liposomes LOrn1 and LOrn3 at the N/P ratios of 1:1, 3:1, 4:1, 6:1 and 8:1. The measurement was carried out in cells by using an inverted fluorescence microscope (10×10). (b) Transfection efficiency in Figure (a) was quantified by flow cytometry analysis.

**Figure S2.** Cell viability assay of HeLa after treating with LOrn1/DNA and LOrn3/DNA lipoplexes for different time by MTT.

**Figure S3.** Small angle x-ray scattering patterns of the lamellar L^C^_α_ and columnar inverted hexagonal H^C^_‖_ phases of (a) LOrn1/DNA and (b) LOrn3/DNA lipoplexes at N/P ratios of 4:1 and 3:1, respectively.

**(b)**

**(a)**

**Figure S4.** Effect of acid environment on lipid structure was detected by ESI-MS. (a) ESI-MS analysis of lipid under the pH of 7.0, (b) ESI-MS analysis of lipid under the pH of 5.5.
